# Different evolutionary trajectories of vaccine-controlled and non-controlled avian infectious bronchitis viruses in commercial poultry

**DOI:** 10.1371/journal.pone.0176709

**Published:** 2017-05-04

**Authors:** Mark W. Jackwood, Dong-Hun Lee

**Affiliations:** 1 Poultry Diagnostic and Research Center, Department of Population Health, College of Veterinary Medicine, University of Georgia, Athens GA, United States of America; 2 Southeast Poultry Research Laboratory, US National Poultry Research Center, ARS, USDA, Athens GA, United States of America; Sun Yat-Sen University, CHINA

## Abstract

To determine the genetic and epidemiological relationship of infectious bronchitis virus (IBV) isolates from commercial poultry to attenuated live IBV vaccines we conducted a phylogenetic network analysis on the full-length S1 sequence for Arkansas (Ark), Massachusetts (Mass) and Delmarva/1639 (DMV/1639) type viruses isolated in 2015 from clinical cases by 3 different diagnostic laboratories. Phylogenetic network analysis of Ark isolates showed two predominant groups linked by 2 mutations, consistent with subpopulations found in commercial vaccines for this IBV type. In addition, a number of satellite groups surrounding the two predominant populations were observed for the Ark type virus, which is likely due to mutations associated with the nature of this vaccine to persist in flocks. The phylogenetic network analysis of Mass-type viruses shows two groupings corresponding to different manufacturers vaccine sequences. No satellite groups were observed for Mass-type viruses, which is consistent with no persistence of this vaccine type in the field. At the time of collection, no vaccine was being used for the DMV/1639 type viruses and phylogenetic network analysis showed a dispersed network suggesting no clear change in genetic distribution. Selection pressure analysis showed that the DMV/1639 and Mass-type strains were evolving under negative selection, whereas the Ark type viruses had evolved under positive selection. This data supports the hypothesis that live attenuated vaccine usage does play a role in the genetic profile of similar IB viruses in the field and phylogenetic network analysis can be used to identify vaccine and vaccine origin isolates, which is important for our understanding of the role live vaccines play in the evolutionary trajectory of those viruses.

## Introduction

Avian Infectious bronchitis virus (IBV) is a gamma coronavirus that causes a highly contagious upper-respiratory tract disease in chickens [1]. Control of IBV in commercial broiler chickens is attempted with attenuated live vaccines. Historically, live vaccines, attenuated by many passages in embryonated eggs, have been used to generate a protective immune response in the upper-respiratory tract. But, some live vaccines can cause a clinical reaction, which often leads producers to give less than a full dose of vaccine, particularly to one-day old chicks. When applied at a full dose per the manufacturer’s instructions, effective vaccines reduce virus replication thereby decreasing the opportunities for mutations and recombination, which can lead to the emergence of new IBV types. However, improper vaccine application or vaccines that do not provide adequate protection due to serotype differences can lead to persistence of viruses in the field and the emergence of new IBV types.

Genetic diversity in avian coronaviruses is created by rapid replication, large population sizes and a high rate of mutations generated from a viral encoded polymerase with limited proofreading capability [2, 3]. There is a plethora of different IBV types, which appears to be unique for this coronavirus, and different IBV types can emerge due to mutations in the spike gene accumulating over time [4]. But the use of attenuated live vaccines, which can be reisolated from chickens, has been shown to cloud the evolutionary picture for viruses in the field [5]. In addition, the use of live vaccines has been shown to increase the mutation rate of IBV presumably by immunologic pressure being exerted on the field viruses [6, 7].

In many poultry producing countries, a combination of vaccines against different IBV types are often given to commercial chickens to provide broader protection against currently circulating strains. However, the emergence of new strains and the reemergence of one or more previously circulating IBV types is unpredictable, which confounds decisions on which combination of vaccines should be used. If the vaccines selected are not effective, outbreaks can occur. Typically, diagnosis and identification of IBV involves reverse transcriptase-polymerase chain reaction (RT-PCR) amplification and sequencing of the S1 subunit of the spike gene or only the hypervariable region of the S1 subunit. The sequence data is then used to report a percent similarity to previously identified isolates. That information is useful to determine the presence of circulating strains in the area, but that data does not provide information on the genetic profile and spread of specific genetic types. Complicating the picture is the persistence of live IBV vaccines in chickens resulting in many of the viruses identified in commercial poultry being closely related or identical to the vaccines being used in the flocks. And, there are no clear and consistent sequence markers in S1 that can be used to definitively distinguish between pathogenic and attenuated viruses.

In this study, we use a molecular epidemiological approach to examine the genetic profile of specific strains of IBV circulating in the field. We conducted a phylogenetic and network analysis on the full-length S1 sequence to determine the genetic relationship, diversity and distribution of three different IBV types (DMV/1639, Ark-DPI and Mass). Network analysis is useful for examining many different nucleic acid sequence data sets, but our work focused on identifying the relationships between genetic subtypes within a strain of IBV circulating in commercial poultry and comparing those subtypes to live IBV vaccines currently being used in those birds. Constructing median-joining networks for individual strains of IBV is important for our understanding of what appears to be unpredictable reemergence and movement of specific IBV types in commercial poultry as well as determining the role attenuated live vaccines play in the genetic makeup of viruses in the field. In addition, we examined the viruses for recombination sequences, single nucleotide polymorphisms and gene-and site-specific selection pressures.

We conducted our analysis on three different IBV types from clinical cases obtained over a 12-month period in 2015. Clinical cases were obtained from 3 different diagnostic laboratories so we could compare virus types circulating in the two-major broiler chicken producing areas on the east coast namely the Delaware/Maryland/Virginia (DELMARVA) peninsula and Georgia. The DELMARVA peninsula was also selected because that is the only area that the recently emerged DMV/1639 variant type of IBV has been found to circulate. The DMV/1639 type of IBV was selected because there was no vaccine available at the time of sample collection. The Mass type of IBV was examined because an effective attenuated live vaccine was being used in those areas, and the Ark type of IBV was selected because it is an example where the attenuated live vaccine being used in those areas does not provide adequate protection against homologous challenge. Previously it has been shown that the Ark-DPI vaccine when given by eye-drop is efficacious; but chicks vaccinated using a hatchery spray cabinet, which is has become standard practice in the industry are not adequately protected against homologous challenge [8]. This appears to be unique to the Ark-DPI vaccine types since other IBV vaccine types given by hatchery spray cabinet are efficacious [8]. Despite this lack of adequate protection, producers refuse to stop using the Ark-DPI vaccine because of the presumed threat of Ark type challenge viruses in the field and because there are no other vaccines commercially available against Ark.

The data generated in this study helps to elucidate the genetic relationships between IB viruses identified in outbreaks of the disease in commercial poultry and contributes to our knowledge on the molecular trajectory of live attenuated vaccine viruses and pathogenic field viruses.

## Materials and methods

### Viruses and sequence information

Allantoic fluids from embryonated chicken eggs containing field isolates of IBV from three different diagnostic laboratories; the Poultry Diagnostic and Research Laboratory, (953 College Station Road, University of Georgia, Athens GA), Georgia Poultry Laboratory, (3235 Abit Massey Way, Gainesville, GA) and the Salisbury Animal Health Laboratory (Maryland Department of Agriculture, Salisbury, MD) were obtained. Virus isolates were previously identified by the diagnostic laboratories using specific real-time RT-PCR or by partial S1 gene sequencing as Arkansas (Ark) 90 isolates, Massachusetts (Mass) 20 isolates and Delmarva/1639 (DMV/1639) 35 isolates. Allantoic fluids from those samples were submitted to our laboratory for virus gene amplification and sequencing. The viruses are from clinical cases which were collected in the year 2015. Isolates with the same number followed by -2 are from different flocks sampled at the same time on the same farm.

Some of the sequences used in this study were obtained from reference strains listed in Table 1.

**Table 1:** Sequences were obtained from the following reference strains.

Strain designation	GenBank Accession Number	Description
Ark/Ark/81	AF006624	Pathogenic virus
Mass/Mass41/41	AY561711	Pathogenic virus
DMV/1639/14	KR232396	Pathogenic virus
GA13/GA13/13	AF419314	Pathogenic virus
PA/171/99	AF419314	Pathogenic virus
PA/Wolgemuth/98	AF305595	Pathogenic virus
Ark A1	EU283045	Vaccine^a^
Ark A2	EU283046	Vaccine
Ark B1	EU283048	Vaccine
Ark B2	EU283049	Vaccine
Ark C1	EU283054	Vaccine
Ark C2	EU283055	Vaccine
Ark A-13	EU283047	Vaccine isolated from vaccinated chickens
Ark B-3	EU283050	Vaccine isolated from vaccinated chickens
Ark B-9	EU283051	Vaccine isolated from vaccinated chickens
Ark C-6	EU283056	Vaccine isolated from vaccinated chickens
Ark B-6	EU283052	Vaccine isolated from contact exposed chickens
Ark B-13	EU283053	Vaccine isolated from contact exposed chickens
Mass A-1	EU283073	Vaccine
Mass A-2	EU283074	Vaccine
Mass B-1	EU283076	Vaccine
Mass B-2	EU283077	Vaccine
Mass C-1	EU283082	Vaccine
Mass D-1	EU283085	Vaccine
Mass D-2	EU283086	Vaccine
Mass A reisol	EU283075	Vaccine isolated from vaccinated chickens
Mass B-3	EU283078	Vaccine isolated from vaccinated chickens
Mass B-9	EU283079	Vaccine isolated from vaccinated chickens
Mass C reisol	EU283084	Vaccine isolated from vaccinated chickens
Mass D-3	EU283087	Vaccine isolated from vaccinated chickens
Mass B-6	EU283080	Vaccine isolated from contact exposed chickens
Mass B-13	EU283081	Vaccine isolated from contact exposed chickens
Mass D-6	EU283088	Vaccine isolated from contact exposed chickens

^a^Sequence obtained from the vaccine in the bottle

### S1 spike gene sequencing and analysis

The S1 spike genes were amplified using the NewS1oligo 5’ (5’–TGA AAC TGA ACA AAA GAC—3') and S1oligo3’ degenerate primers (5’–CCA TAA GTA ACA TAA GGR CRA—3') [9] together with the Titan One Tube RT-PCR kit (Roche) as previously described [9]. Samples were sent to the Georgia Genomics Facility (Athens, GA) for standard Sanger sequencing using the NewS1oligo 5’ and S1oligo3’deg. The spike gene for each sample was sequenced and assembled using SeqBuilder software, Version 13.0 (DNAStar Inc., Madison WI).

### Analysis of recombination

Putative recombinant sequences were identified with the software package of SimPlot (SCRoftware, Baltimore MD). The nucleotide sequences of S1 gene based on the multiple alignment results were introduced into similarity plots with SimPlot version 3.5.1. The nucleotide identity was calculated using the Kimura 2-parameter method with a transition–transversion ratio of 2 in each window of 200 bp with a step size of 20 bp.

### Phylogenetic analysis

Coding regions of the S1 gene were aligned using Multiple Alignment with Fast Fourier Transformation (MAFFT) in Geneious v8.1.2 program [10]. The best-fit nucleotide substitution model was determined and the ML tree was constructed by the MEGA 6 software [11] using the Hasegawa-Kishino-Yano model of nucleotide substitution with gamma-distributed rate variation among sites (with four rate categories) with subtree pruning and regrafting branch swapping. Statistical support for the tree topology was determined by bootstrap analysis with 1000 replicates. The phylogenetic network tree was constructed using the Median Joining method implemented in NETWORK ver. 5.0 [12]. The phylogenetic network included the most parsimonious trees linking the sequences. Each unique sequence is represented by a circle whose size reflects the frequency of the sequence in the data set. Branch length is proportional to the number of mutations.

### Analysis of selection pressures

Gene- and site-specific selection pressures for the S1 protein of the IBVs were measured as the ratio of non-synonymous (dN) to synonymous (dS) nucleotide substitutions per site. The dN/dS ratios and the selection pressures at individual codons were estimated using the single-likelihood ancestor counting (SLAC), fixed-effects likelihood (FEL), Internal fixed-effects Likelihood (IFEL), random effects likelihood (REL), Fast Unconstrained Bayesian Approximation for Inferring Selection (FUBAR), and mixed effects model of episodic diversifying selection (MEME) available at the DataMonkey online version of the HY-Phy package (http://www.datamonkey.org). All analyses utilized the best-fit model of nucleotide substitution and employed input NJ phylogenetic trees. Positively selected sites that were confirmed by at least two different methods were included in this study. In silico N-linked glycosylation prediction was performed using GlycoEP [13].

## Results

Isolate designations and GenBank accession numbers are shown in S1 Table (available in the online Supplementary Material).

### Recombination analysis

**Fig 1:**
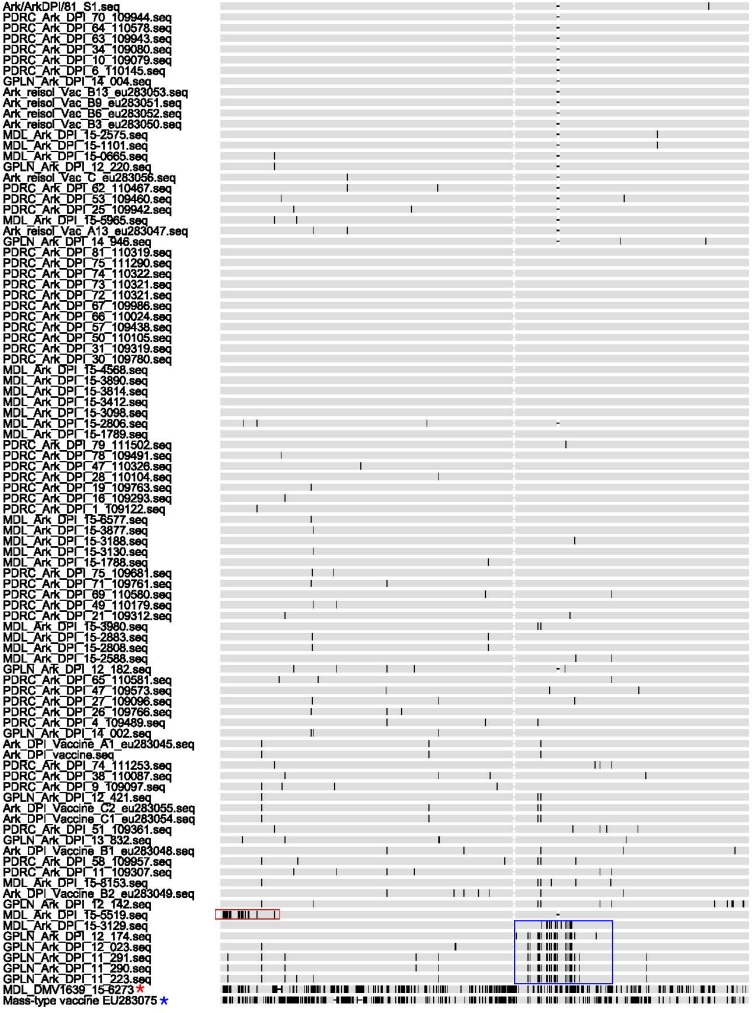
Single nucleotide polymorphisms (SNPs) for Ark viruses. Red box = recombination of MDL-Ark-DPI-15-5519 with DMV/1639 (24 SNPs in the first 110nt), and the Blue boxes = recombination of 6 Ark strains with Mass vaccine type virus.

Ark sequences were examined for recombination and analysis of the data (Fig 1) shows that one sequence MDL-Ark-DPI-15-5519 recombined with a DMV/1639 type virus (24 SNPs in the first 110 nt, red box) and six viruses GPLN-Ark-DPI-11-290, GPLN-Ark-DPI-11-291, GPLN-Ark-DPI-11-223, GPLN-Ark-DPI-12-174, GPLN-Ark-DPI-12-023 and MDL-Ark-DPI-15-3129 recombined with a Mass-type virus (9 to 16 SNPs between nt 960 and nt 1100, blue boxes). DMV/1639 and Mass type isolates were not found to have recombination with other IBV types.

### Phylogenetic analysis

**Fig 2:**
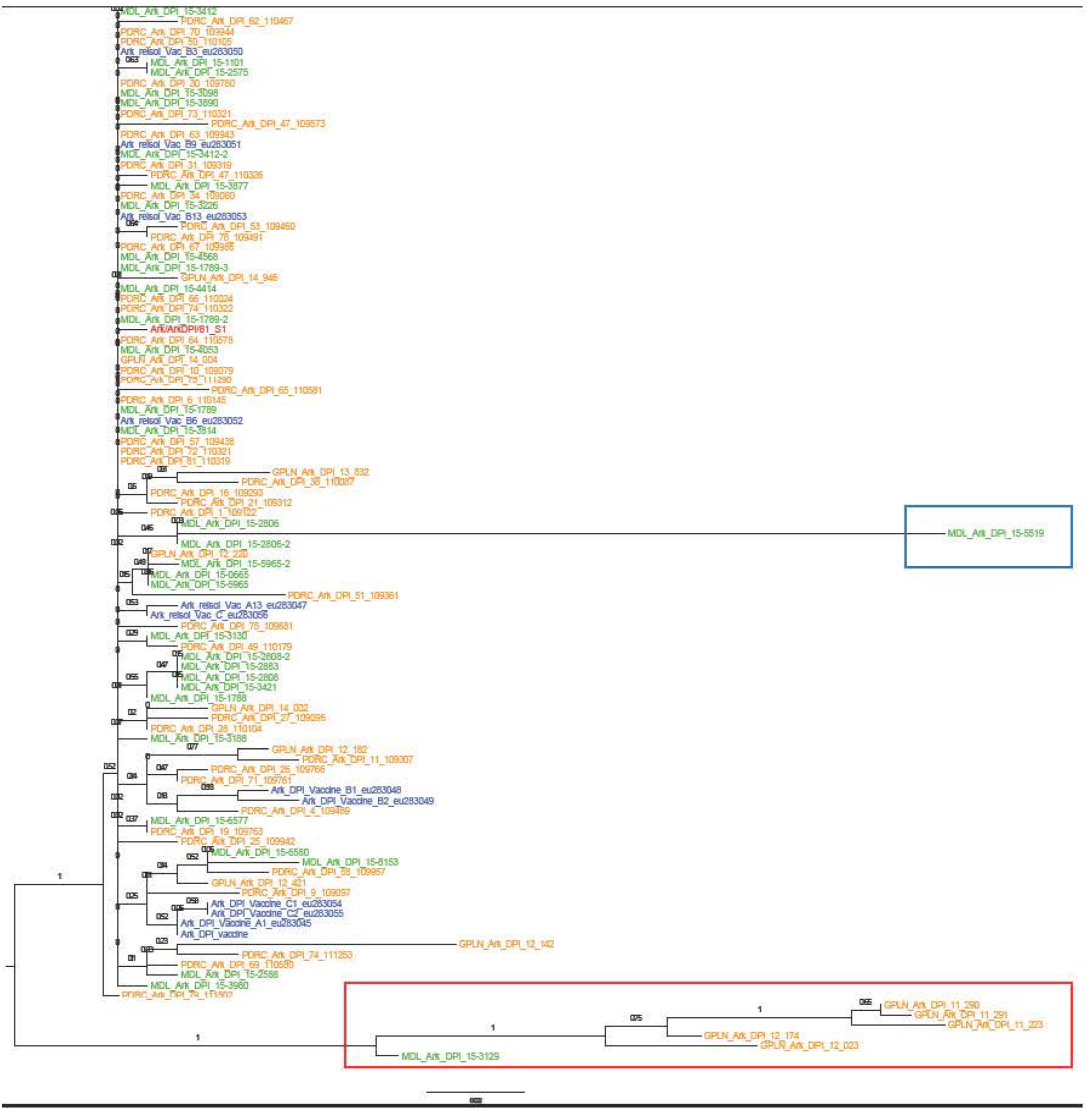
Phylogenetic tree created by the Maximum-likelihood algorithm for the S1 gene of IBV Ark type viruses. Red is pathogenic Ark/Ark-DPI/81, blue is vaccine virus, green is viruses from DELMARVA and yellow is viruses from Georgia. Blue box = recombined with DMV/1639/14 and red box = recombined with Mass type virus.

**Fig 3:**
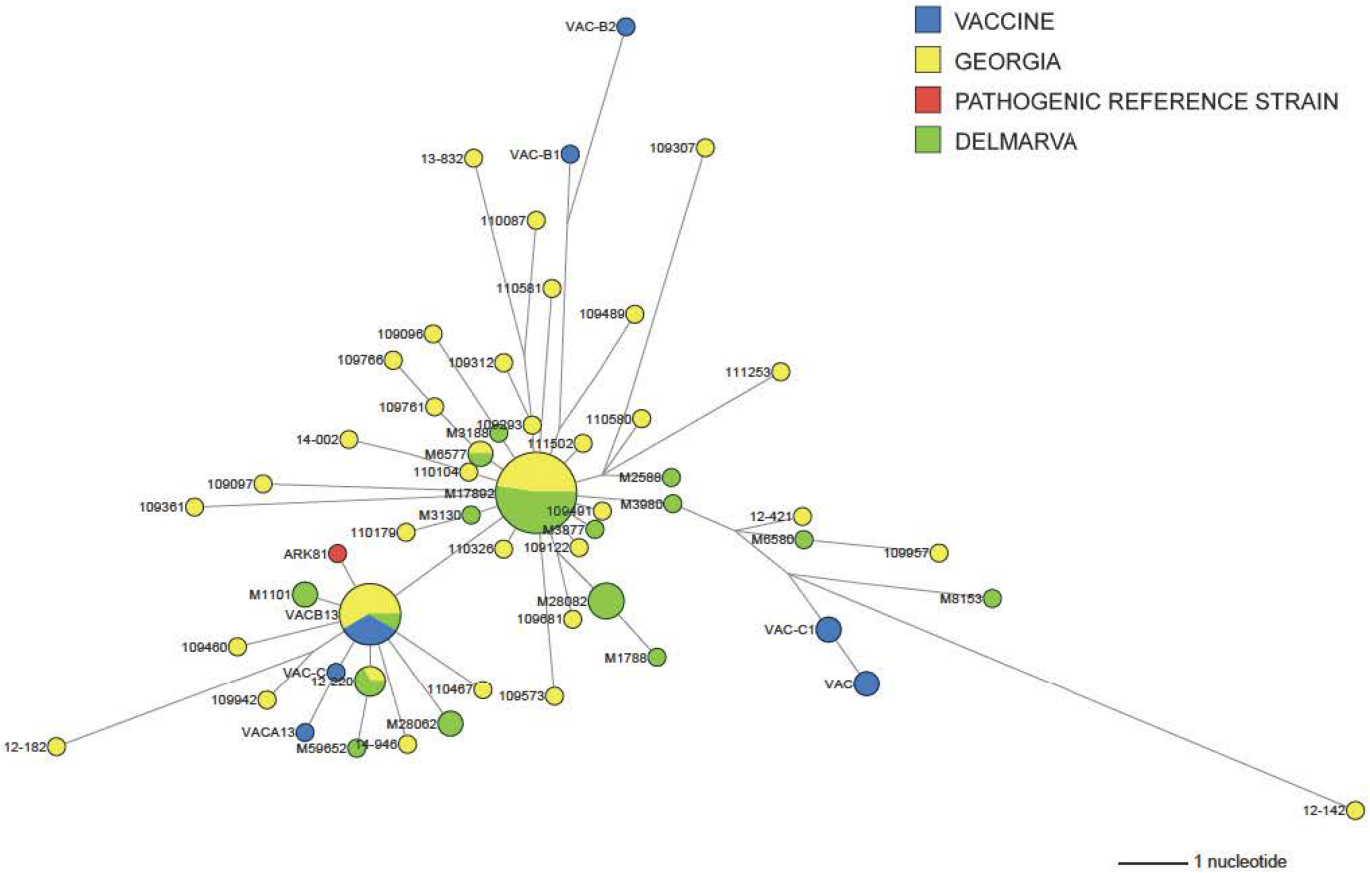
Median-joining phylogenetic network of Ark type viruses. The median-joining network was constructed from the S1-encoding gene. The network included the most parsimonious trees linking the sequences. Viruses with identified recombination sequences to DMV/1639 or Mass-type viruses were not included in the analysis. Each unique sequence is represented by a circle whose size reflects the frequency of the sequence in the data set. Branch length is proportional to the number of mutations. Isolates are colored according to their origin as follows: blue = vaccine, green = DELMARVA, yellow = Georgia and red = pathogenic reference strain.

The Ark taxon showed a single phylogeny with outliers being viruses that recombined with either DMV/1639 or Mass (Fig 2). The Ark viruses with identified recombination sequences to DMV/1639 or Mass-type viruses were not included in the network analysis. Phylogenetic network analysis of Ark type viruses (Fig 3) clearly showed the presence of two predominant groups; one major population containing 21 taxa represented by M17892 and a second population containing 12 taxa represented by VAC-B13. The two groups are separated from each other by 3 mutations. Viruses in both groups are experimentally reisolated Ark-Delmarva Poultry Industry (Ark-DPI) vaccine viruses as well as Ark-DPI vaccine-like field isolates. The Ark-DPI vaccine sequences obtained from GenBank (Ark-A1, Ark-A2, Ark-B1, Ark-B2, Ark-C1 and Ark-C2) are not observed among the field isolates but linked to the largest group that contains 21 taxa.

**Fig 4:**
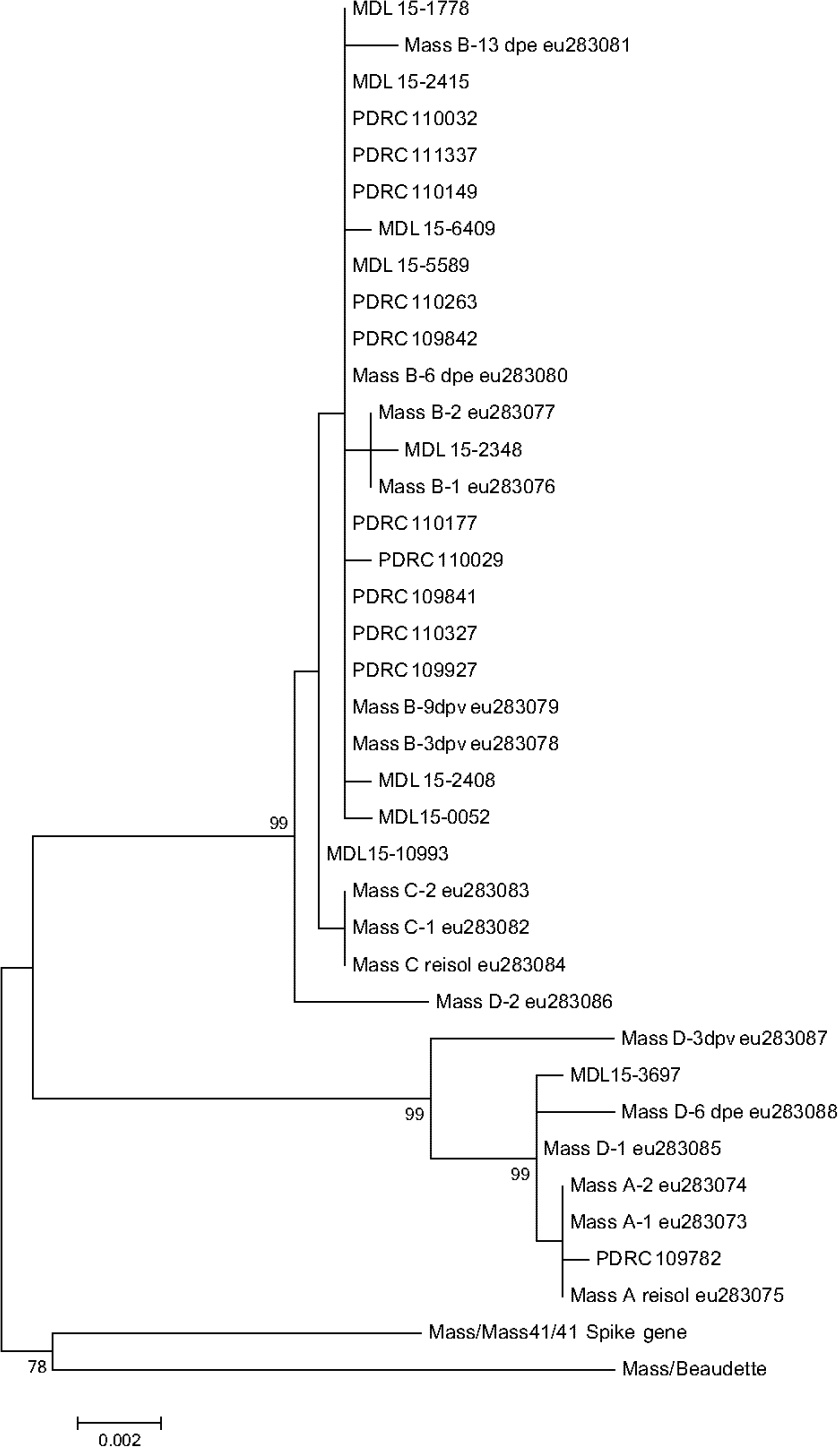
Phylogenetic tree created by the Maximum-likelihood algorithm for the S1 gene of IBV Mass type viruses.

**Fig 5:**
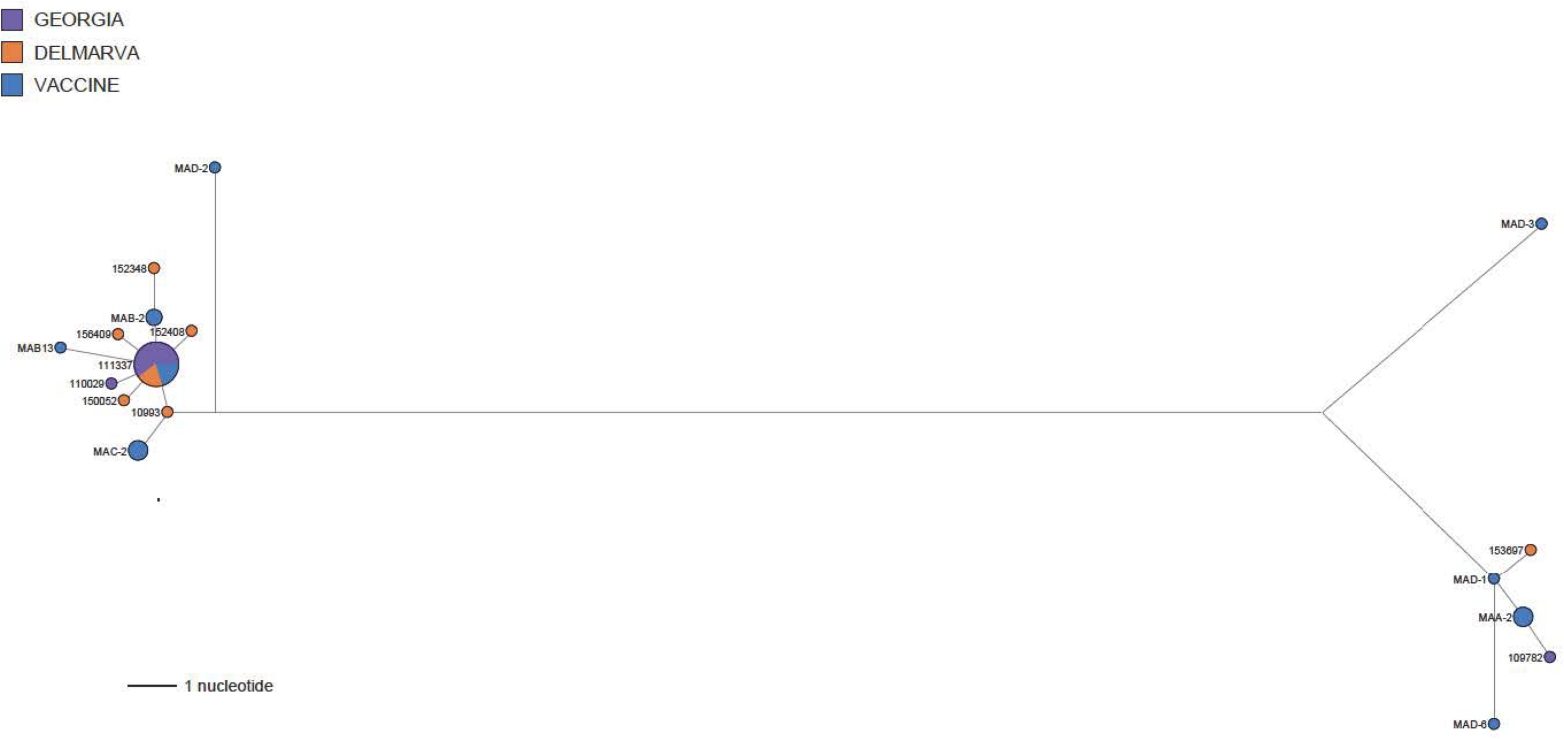
Median-joining phylogenetic network of Mass-type viruses. The median-joining network was constructed from the S1-encoding gene. The network included the most parsimonious trees linking the sequences. Each unique sequence is represented by a circle whose size reflects the frequency of the sequence in the data set. Branch length is proportional to the number of mutations. Isolates are colored according to their origin as follows: blue = vaccine, orange = DELMARVA, purple = Georgia.

The maximum likelihood (ML) phylogenetic tree for Mass-type viruses showed at least 3 groups supported by bootstrap values > 70%: Group 1, the largest group (27 isolates) related to Mass B and Mass C type vaccines (bootstrap value of 99%); Group 2, vaccine type viruses related to Mass A type vaccines (8 isolates) (bootstrap value of 99%); Group 3, the pathogenic Mass/Mass41/41 reference virus and laboratory Beaudette strain (bootstrap value of 78%) (Fig 4). Consistent with ML phylogenetic analysis, the phylogenetic network analysis of Groups 1 and 2 Mass vaccine type viruses (Fig 5) shows two groupings linked by a long branch. Group 1 consists of 27 taxon including B and C type vaccines and field isolates form DELMARVA and GA. Group 2 consists of 8 taxon including A and D type vaccines and a field isolate from DELMARVA and one from GA.

**Fig 6:**
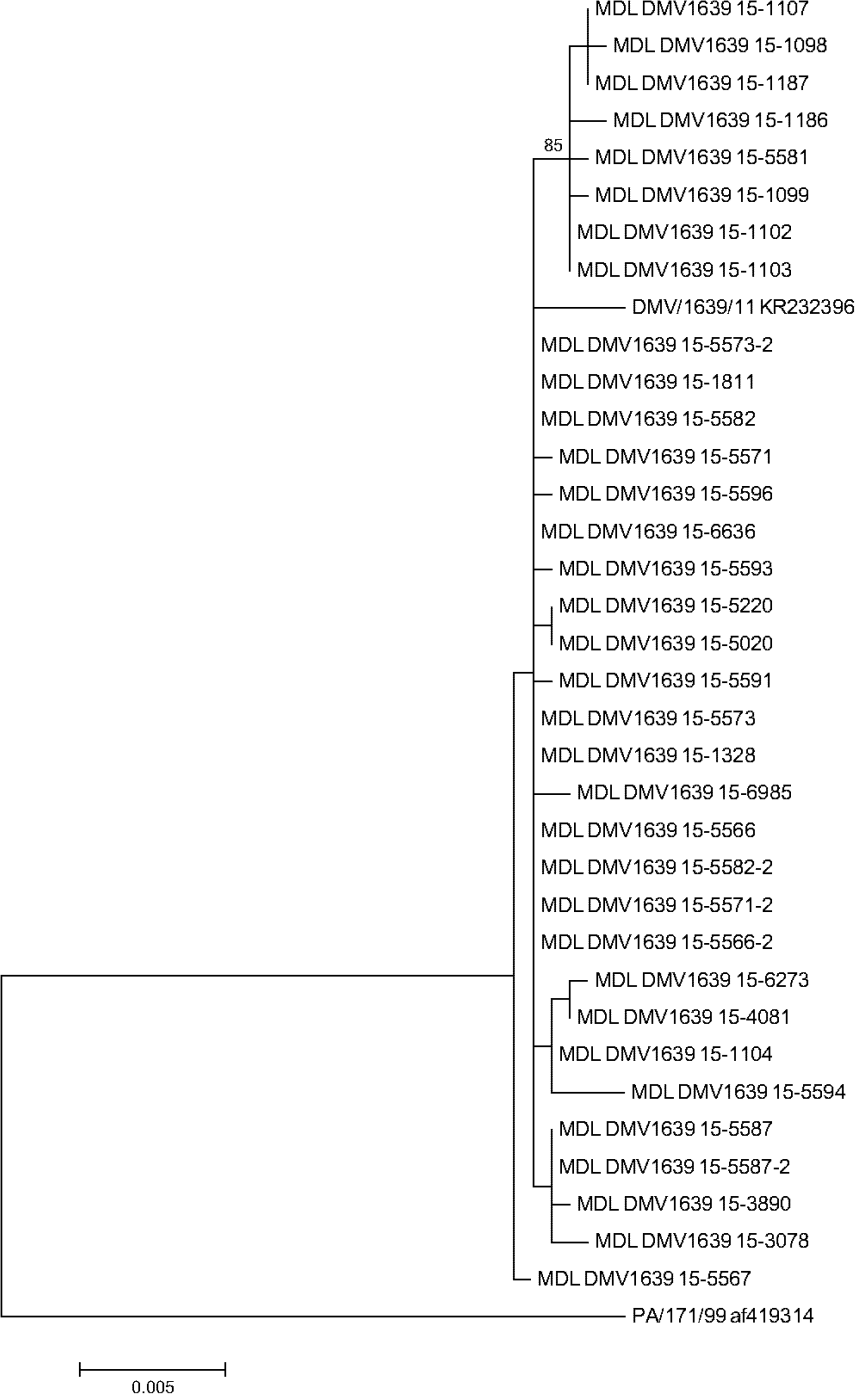
Phylogenetic tree created by the Maximum-likelihood algorithm for the S1 gene of IBV DMV/1639 type viruses.

**Fig 7:**
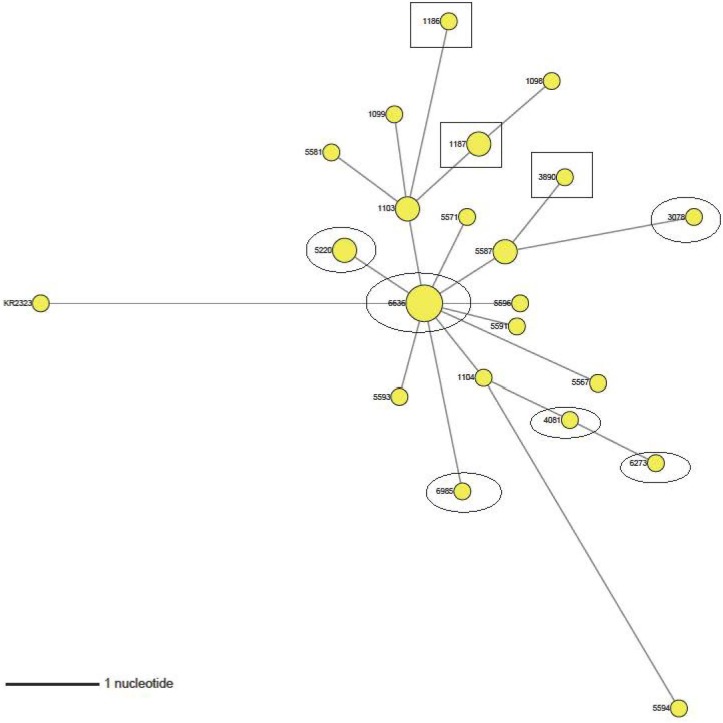
Median-joining phylogenetic network of DMV/1639 viruses. The median-joining network was constructed from the S1-encoding gene. The network included the most parsimonious trees linking the sequences. Each unique sequence is represented by a circle whose size reflects the frequency of the sequence in the data set. Branch length is proportional to the number of mutations. Sequences with a rectangle around them are from nephropathogenicity cases. Sequences with an oval around them are from cases with respiratory signs only. All other sequences were from cases where the disease presentation was not known. All of the viruses are from DELMARVA.

The ML tree for DMV/1639 showed a narrow monophyletic topology that was distinct from the PA/171/99 reference virus (Fig 6). The S1 gene of the PA/171/99 virus was used as a reference because it has 95.8% identity with DMV/1639 and is thought to be the progenitor of that virus (Jackwood, unpublished data). Consistent with ML analysis, the phylogenetic network analysis of DMV/1639 (Fig 7) has a more widely distributed network appearance with no clear groupings. It should be noted that all of the DMV/1639 isolates came from the Salisbury Animal Health Laboratory since that strain of IBV is currently only circulating on the DELMARVA peninsula. The earliest isolate of DMV/1639 obtained in 2015 was 1098 and the most recent isolate was 6985. Examining the pathogenicity of the isolates in relationship to the network shows that early isolates (1186, 1187 and 3890) are associated with cases of nephropathogenicity (rectangles in Fig 7), whereas later isolates (3078, 4081, 5220, 5573, 6273, 6636, and 6985) are from cases with only respiratory signs (ovals in Fig 7).

### Analysis of selection pressures

We analyzed the selection profiles of the S1 protein of Mass, DMV, and Ark strains. The dN/dS ratio of Mass and DMV strains were 0.681 and 0.503, respectively, indicating that the S1 region of the IBV genome of these strains had evolved under negative selection (Table 2). However, the dN/dS ratio of the Ark strain was 1.230, indicating that this strain had evolved under positive selection. We found ten individual codons (p-value<0.1) that may be under positive selection in the S1 protein of the Ark strain, three putatively positive selected residues in the Mass strain (p-value<0.1) and one in the DMV (p-value<0.1) strain. There were three positively selected residues (56, 323 and 386) in Ark type viruses that were previously reported for antigenic escape mutants [14, 15]. There were 18 potential glycosylation sites for Ark type and Mass type viruses, whereas DMV/1639 type viruses had 19 potential glycosylation sites. None of the sites were found to contain positively selected residues.

**Table 2:** Selection profiles of the S1 spike protein.

Type	No. of sequences	Mean dN/dS	Positively selected sites^a^	
			N	Amino acid position ^b^
Mass	27	0.681	3	129 (N/H), 179(E/A), 273(T/S)
DMV	35	0.503	1	90 (G/D/N/S)
Ark	96	1.230	10	56^c^ (N/S), 94 (A/T/V), 119 (S/P), 171 (H/Y), 223 (H/Q/Y), 323^c^ (R/T/M), 326 (N/Y), 362 (Q/R), 386^c^ (R/H/L), 399 (H/Y)

^a^None of the sites are potential glycosylation sites

^b^ Amino acid positions correspond to the S1 spike protein of each type.

^c^ Sites for previously reported antigenic escape mutants

## Discussion

In this study, we conducted molecular epidemiological analysis on IBV types circulating in commercial poultry over a 1 year period. To understand the effect of live attenuated vaccines on IBV isolates from chickens in the field, we chose to analyze 3 different IBV types. The Ark type of IBV was selected because it is an example of an IBV type where the vaccine does not provide adequate protection against homologous challenge. The Mass type of IBV was selected as an example of an IBV type where an effective vaccine is widely used. And finally, the DMV/1639 type of IBV was selected because no vaccine was available for that virus type at the time of sample collection.

Previously it has been shown that the Ark-DPI live attenuated vaccine strain when given using a hatchery spray cabinet does not protect chickens against homologous challenge [8]. In addition, that vaccine has been shown to persist in chicks sometimes resulting in a ‘rolling’ vaccine reaction in the flock. Rolling vaccine reactions occur when a small proportion of the flock becomes infected with the vaccine then transmits that vaccine to susceptible flock mates, which results in back passage of the vaccine and ultimately vaccine reactions in the flock. This is unique to the ArkDPI vaccine and is due to a minor subpopulation in the vaccine, exhibiting polymorphisms in the S1 spike, being the most fit population for infecting the bird; whereas the major subpopulation in the vaccine, which is well suited for vaccine propagation in embryonating eggs, is not very well suited for infection in chicks [16]. Consequently, only a small amount of the most fit virus subpopulation reaches the chick even when a full dose of vaccine is administered. The result of persisting Ark-DPI vaccine in the flock is that it is extremely difficult to determine if pathogenic virus is still circulating in the field.

Recombination between heterologous strains of IBV has been shown in the field [17]. Our sequence analysis showed one Ark isolate had recombined with DMV/1639 and six Ark isolates recombined with Mass-type viruses in the S1 gene. The wide spread usage and continued replication of the Ark-DPI vaccine in the field undoubtedly provides opportunities for recombination events. Additionally, common use of Mass-type vaccines, sometimes given at the same time as Ark-DPI vaccines, can explain why more recombinations between Ark and Mass-type viruses were observed compared to recombinations between Ark and DMV/1639 viruses. Additionally, DMV/1639 is not widespread, only circulating on the DELMARVA peninsula, which would also limit the opportunity for DMV/1639 to recombine with other IBV types.

Phylogenetic network analysis of the Ark type viruses showed two predominant groups linked by 2 mutations with many satellite groups surrounding. The existence of experimentally reisolated Ark-DPI vaccine viruses and Ark-DPI vaccine-like field viruses in the predominant groups is consistent with the presence of minor subpopulations in the vaccine vials that are the most fit viruses to infect and replicate in chickens. Consensus sequences obtained directly from the vaccine bottle are observed in the network analysis as satellite populations with no field viruses included. The number of individual viruses or small groups of virus types around each of the two predominant clusters suggests that these satellite groups are the result of mutations occurring in Ark-DPI vaccine viruses persisting in the flocks. This is consistent with a higher dN/dS ratio for Ark-DPI (1.230) compared to DMV/1639 (0.503) and Mass/Mass41/41 (0.681), which likely reflects the replication and subsequent adaptation of Ark-DPI vaccine virus persisting in chickens.

ML phylogenetic and network analysis of the Mass-type viruses showed two vaccine related groups and outgroup containing pathogenic Mass/Mass41/41 and the laboratory strain Beaudette. Group 1 contains taxon from vaccine reference strains designated B and C and group 2 contains taxon from vaccine reference strains A and D. Field isolates within each group suggest that these reisolated viruses are vaccine in origin and not pathogenic viruses related to Mass/Mass41/41. Mass type vaccines do not persist in the field like Ark-DPI vaccines, thus satellite groups around a larger group might not be observed.

ML phylogenetic and network analysis of the DMV/1639 viruses showed a widely dispersed network suggesting no clear mutational direction. There was no vaccine available for the DMV/1639 type viruses at the time our samples were taken, which may have played a role in the dispersed network pattern observed. Examining the pathogenicity associated with isolates of DMV/1639 showed that early isolates were associated with nephropathogenicity whereas later isolates were primarily associated with respiratory only clinical signs. This is consistent with observations in the field showing that the pathogenicity of DMV/1639 has progressed from nephropathogenic to primarily respiratory in nature (Personnel Communication Dr. Erin Brannick, University of Delaware, Newark DE). Adaptation from a highly virulent virus causing lesions in the kidney that can result in significant mortality to a virus causing only mild respiratory signs and little or no mortality in the host is consistent with our observation that DMV/1639 is evolving under negative pressure.

The viruses in this study that were obtained from diagnostic laboratory submissions all came from samples collected from birds experiencing disease. When live attenuated vaccines are used in commercial poultry flocks, the virus type isolated, even from diseased birds is often the same type as the vaccine. The question then remains if the isolated virus is attenuated vaccine masking the real problem virus, vaccine virus that has been circulating in the field and has reverted to pathogenicity, or a truly pathogenic field virus that has broken through the immune response of the bird. Examining our network analysis data for Mass, we found that the Mass viruses isolated were indeed grouped with attenuated vaccine viruses. Since IBV vaccines are adapted to grow well in embryonated eggs, the vaccine strain will often outgrow a pathogenic field virus when they are in the same sample, leading to identification of only the vaccine virus. However, network analysis of the Ark viruses shows them to be vaccine origin viruses that have undergone selection and mutations indicating that they have been circulating for some time in the field. Passage from bird to bird can serve to “heat up” the vaccine virus resulting in reversion to pathogenicity, thus potentially explaining the clinical signs observed in the flock. This observation is significant because it shows for the first time that the clinical signs of disease associated with the Ark type of IBV in commercial poultry are resulting from usage of the vaccine.

In summary, constructing ML phylogenetic tree and median-joining networks for individual strains of IBV with vaccines in use (Ark and Mass) showed considerably different evolutionary trajectories compared to a virus type (DMV/1639) where no vaccine was in use at the time. This data supports the hypothesis that the predominant groups of the Ark-DPI vaccine like viruses found in the field originated from vaccine viruses. Thus, it appears that live attenuated vaccine usage does play a role in the genetic profile of similar viruses in the field and phylogenetic network analysis can be used to identify vaccine and vaccine origin isolates, which is important for our understanding of the epidemiology of IBV.

## Supporting information

S1 Table Virus isolates sequenced in this manuscript and GenBank accession numbers. (DOCX)

## References

[1] Jackwood MW, de Wit S. Infectious Bronchitis In: Swayne DE, Glisson JR, McDougald LR, Nolan LK, Suarez DL, Nair V, editors. Diseases of Poultry. 13 ed: John Wiley and Sons, Inc.; 2013 p. 139–59.

[2] Minskaia E, Hertzig T, Gorbalenya AE, Campanacci V, Cambillau C, Canard B, et al Discovery of an RNA virus 3'->5' exoribonuclease that is critically involved in coronavirus RNA synthesis. Proc Natl Acad Sci U S A. 2006;103(13):5108–13. Epub 2006/03/22. doi: 10.1073/pnas.0508200103 16549795 10.1073/pnas.0508200103 PMC1458802

[3] Gorbalenya AE, Enjuanes L, Ziebuhr J, Snijder EJ. Nidovirales: evolving the largest RNA virus genome. Virus Res. 2006;117(1):17–37. Epub 2006/03/01. doi: 10.1016/j.virusres.2006.01.017 16503362 10.1016/j.virusres.2006.01.017 PMC7114179

[4] Holmes EC. The evolutionary genetics of emerging viruses. Annual Review of Ecology Evolution and Systematics. 2009;40:353–72.

[5] McKinley ET, Hilt DA, Jackwood MW. Avian coronavirus infectious bronchitis attenuated live vaccines undergo selection of subpopulations and mutations following vaccination. Vaccine. 2008;26(10):1274–84. doi: 10.1016/j.vaccine.2008.01.006 18262691 10.1016/j.vaccine.2008.01.006 PMC7115600

[6] Jackwood MW, Hall D, Handel A. Molecular evolution and emergence of avian gammacoronaviruses. Infection, genetics and evolution: journal of molecular epidemiology and evolutionary genetics in infectious diseases. 2012;12(6):1305–11. doi: 10.1016/j.meegid.2012.05.003 22609285 10.1016/j.meegid.2012.05.003 PMC7106068

[7] Lee CW, Jackwood MW. Origin and evolution of Georgia 98 (GA98), a new serotype of avian infectious bronchitis virus. Virus Res. 2001;80(1–2):33–9. 11597746 10.1016/s0168-1702(01)00345-8

[8] Roh HJ, Hilt D, Williams SM, Jackwood MW. Evaluation of infectious bronchitis virus Arkansas-type vaccine failrue in commercial broilers. Avian Diseases. 2013;57:248–59. doi: 10.1637/10459-112812-Reg.1 24689182 10.1637/10459-112812-Reg.1

[9] Lee CW, Hilt DA, Jackwood MW. Redesign of primer and application of the reverse transcriptase-polymerase chain reaction and restriction fragment length polymorphism test to the DE072 strain of infectious bronchitis virus. Avian Dis. 2000;44(3):650–4. 11007014

[10] Katoh K, Standley DM. MAFFT multiple sequence alignment software version 7: improvements in performance and usability. Mol Biol Evol. 2013;30(4):772–80. doi: 10.1093/molbev/mst010 23329690 10.1093/molbev/mst010 PMC3603318

[11] Tamura K, Stecher G, Peterson D, Filipski A, Kumar S. MEGA6: Molecular Evolutionary Genetics Analysis version 6.0. Mol Biol Evol. 2013;30(12):2725–9. doi: 10.1093/molbev/mst197 24132122 10.1093/molbev/mst197 PMC3840312

[12] Bandelt HJ, Forster P, Rohl A. Median-joining networks for inferring intraspecific phylogenies. Mol Biol Evol. 1999;16(1):37–48. 10331250 10.1093/oxfordjournals.molbev.a026036

[13] Chauhan JS, Rao A, Raghava GP. In silico platform for prediction of N-, O- and C-glycosites in eukaryotic protein sequences. PLoS One. 2013;8(6):e67008 doi: 10.1371/journal.pone.0067008 23840574 10.1371/journal.pone.0067008 PMC3695939

[14] Kant A, Koch G, van Roozelaar DJ, Kusters JG, Poelwijk FA, van der Zeijst BA. Location of antigenic sites defined by neutralizing monoclonal antibodies on the S1 avian infectious bronchitis virus glycopolypeptide. J Gen Virol. 1992;73(Pt 3):591–6. 1372036 10.1099/0022-1317-73-3-591

[15] Moore KM, Jackwood MW, Hilt DA. Identification of amino acids involved in a serotype and neutralization specific epitope within the s1 subunit of avian infectious bronchitis virus. Archives of virology. 1997;142(11):2249–56. 9672590 10.1007/s007050050239 PMC7087143

[16] Leyson C, Franca M, Jackwood M, Jordan B. Polymorphisms in the S1 spike glycoprotein of Arkansas-type infectious bronchitis virus (IBV) show differential binding to host tissues and altered antigenicity. Virology. 2016;498:218–25. doi: 10.1016/j.virol.2016.08.030 27619927 10.1016/j.virol.2016.08.030 PMC7111678

[17] Lim TH, Lee HJ, Lee DH, Lee YN, Park JK, Youn HN, et al An emerging recombinant cluster of nephropathogenic strains of avian infectious bronchitis virus in Korea. Infection, genetics and evolution. 2011;11(3):678–85. Epub 2011/01/25. doi: 10.1016/j.meegid.2011.01.007 21255688 10.1016/j.meegid.2011.01.007 PMC7185786

