# Supplementary material for: Different evolutionary trajectories of vaccine-controlled and non-controlled avian infectious bronchitis viruses in commercial poultry
Source: PLoS One. 2017 May 4;12(5):e0176709. doi: 10.1371/journal.pone.0176709 (PMC5417570; doi:10.1371/journal.pone.0176709)
Supplement: S1 Table — (DOCX) [file pone.0176709.s001.docx]

Table S1. Virus isolates sequenced in this manuscript and GenBank accession numbers.

Isolate Designation GenBank Accession Number

PDRC_111337.seq KX529700

PDRC_110327.seq KX529701

PDRC_110263.seq KX529702

PDRC_110177.seq KX529703

PDRC_110149.seq KX529704

PDRC_110032.seq KX529705

PDRC_110029.seq KX529706

PDRC_109927.seq KX529707

PDRC_109842.seq KX529708

PDRC_109841.seq KX529709

PDRC_109782.seq KX529710

MDL15-10993.seq KX529711

MDL15-3697.seq KX529712

MDL15-0052.seq KX529713

MDL_15-6409.seq KX529714

MDL_15-5589.seq KX529715

MDL_15-2415.seq KX529716

MDL_15-2408.seq KX529717

MDL_15-2348.seq KX529718

MDL_15-1778.seq KX529719

MDL_DMV1639_15-6636.seq KX529720

MDL_DMV1639_15-6273.seq KX529721

MDL_DMV1639_15-5596.seq KX529722

MDL_DMV1639_15-5591.seq KX529723

MDL_DMV1639_15-5587.seq KX529724

MDL_DMV1639_15-5582.seq KX529725

MDL_DMV1639_15-5573.seq KX529726

MDL_DMV1639_15-5571.seq KX529727

MDL_DMV1639_15-5567.seq KX529728

MDL_DMV1639_15-5566.seq KX529729

MDL_DMV1639_15-5220.seq KX529730

MDL_DMV1639_15-4081.seq KX529731

MDL_DMV1639_15-3890.seq KX529732

MDL_DMV1639_15-1811.seq KX529733

MDL_DMV1639_15-1328.seq KX529734

MDL_DMV1639_15-1187.seq KX529735

MDL_DMV1639_15-1186.seq KX529736

MDL_DMV1639_15-1107.seq KX529737

MDL_DMV1639_15-1104.seq KX529738

MDL_DMV1639_15-1103.seq KX529739

MDL_DMV1639_15-1102.seq KX529740

MDL_DMV1639_15-1099.seq KX529741

GPLN_Ark_DPI_14_946.seq KX529742

GPLN_Ark_DPI_14_002.seq KX529743

GPLN_Ark_DPI_13_832.seq KX529744

GPLN_Ark_DPI_12_421.seq KX529745

GPLN_Ark_DPI_12_220.seq KX529746

GPLN_Ark_DPI_12_182.seq KX529747

GPLN_Ark_DPI_12_174.seq KX529748

GPLN_Ark_DPI_12_142.seq KX529749

GPLN_Ark_DPI_12_023.seq KX529750

GPLN_Ark_DPI_11_291.seq KX529751

GPLN_Ark_DPI_11_290.seq KX529752

GPLN_Ark_DPI_11_223.seq KX529753

GPLN_Ark_DPI_14_004.seq KX529754

MDL_Ark_DPI_15-8153.seq KX529755

MDL_Ark_DPI_15-6577.seq KX529756

MDL_Ark_DPI_15-5965.seq KX529757

MDL_Ark_DPI_15-5519.seq KX529758

MDL_Ark_DPI_15-4568.seq KX529759

MDL_Ark_DPI_15-3980.seq KX529760

MDL_Ark_DPI_15-3890.seq KX529761

MDL_Ark_DPI_15-3877.seq KX529762

MDL_Ark_DPI_15-3814.seq KX529763

MDL_Ark_DPI_15-3412.seq KX529764

MDL_Ark_DPI_15-3188.seq KX529765

MDL_Ark_DPI_15-3130.seq KX529766

MDL_Ark_DPI_15-3129.seq KX529767

MDL_Ark_DPI_15-3098.seq KX529768

MDL_Ark_DPI_15-2883.seq KX529769

MDL_Ark_DPI_15-2808.seq KX529770

MDL_Ark_DPI_15-2806.seq KX529771

MDL_Ark_DPI_15-2588.seq KX529772

MDL_Ark_DPI_15-2575.seq KX529773

MDL_Ark_DPI_15-1789.seq KX529774

MDL_Ark_DPI_15-1788.seq KX529775

MDL_Ark_DPI_15-1101.seq KX529776

MDL_Ark_DPI_15-0665.seq KX529777

PDRC_Ark_DPI_66_110024.seq KX529778

PDRC_Ark_DPI_65_110581.seq KX529779

PDRC_Ark_DPI_64_110578.seq KX529780

PDRC_Ark_DPI_63_109943.seq KX529781

PDRC_Ark_DPI_62_110467.seq KX529782

PDRC_Ark_DPI_58_109957.seq KX529783

PDRC_Ark_DPI_57_109438.seq KX529784

PDRC_Ark_DPI_53_109460.seq KX529785

PDRC_Ark_DPI_51_109361.seq KX529786

PDRC_Ark_DPI_50_110105.seq KX529787

PDRC_Ark_DPI_49_110179.seq KX529788

PDRC_Ark_DPI_47_110326.seq KX529789

PDRC_Ark_DPI_47_109573.seq KX529790

PDRC_Ark_DPI_38_110087.seq KX529791

PDRC_Ark_DPI_34_109080.seq KX529792

PDRC_Ark_DPI_31_109319.seq KX529793

PDRC_Ark_DPI_30_109780.seq KX529794

PDRC_Ark_DPI_28_110104.seq KX529795

PDRC_Ark_DPI_27_109096.seq KX529796

PDRC_Ark_DPI_26_109766.seq KX529797

PDRC_Ark_DPI_25_109942.seq KX529798

PDRC_Ark_DPI_21_109312.seq KX529799

PDRC_Ark_DPI_19_109763.seq KX529800

PDRC_Ark_DPI_16_109293.seq KX529801

PDRC_Ark_DPI_11_109307.seq KX529802

PDRC_Ark_DPI_10_109079.seq KX529803

PDRC_Ark_DPI_9_109097.seq KX529804

PDRC_Ark_DPI_6_110145.seq KX529805

PDRC_Ark_DPI_4_109489.seq KX529806

PDRC_Ark_DPI_1_109122.seq KX529807

PDRC_Ark_DPI_81_110319.seq KX529808

PDRC_Ark_DPI_79_111502.seq KX529809

PDRC_Ark_DPI_78_109491.seq KX529810

PDRC_Ark_DPI_75_111290.seq KX529811

PDRC_Ark_DPI_75_109681.seq KX529812

PDRC_Ark_DPI_74_111253.seq KX529813

PDRC_Ark_DPI_74_110322.seq KX529814

PDRC_Ark_DPI_73_110321.seq KX529815

PDRC_Ark_DPI_72_110321.seq KX529816

PDRC_Ark_DPI_71_109761.seq KX529817

PDRC_Ark_DPI_70_109944.seq KX529818

PDRC_Ark_DPI_69_110580.seq KX529819

PDRC_Ark_DPI_67_109986.seq KX529820
